# Dexmedetomidine in the Treatment of Depression: An Up-to-date Narrative Review

**DOI:** 10.2174/17450179-v19-230823-2023-4

**Published:** 2023-08-30

**Authors:** Tamadhir Al-Mahrouqi, Mohammed Al Alawi, Rafael C. Freire

**Affiliations:** 1 Department of Behavioural Medicine, Sultan Qaboos University Hospital, Muscat, Oman; 2 Psychiatry Residency Training Program, Oman Medical Speciality Board, Muscat, Oman; 3 Department of Psychiatry and Centre for Neuroscience Studies, Queens University, Kingston, Canada; 4 Laboratory of Panic and Respiration, Institute of Psychiatry, Federal University of Rio de, Janeiro, Rio de Janeiro, Brazil

**Keywords:** Dexmedetomidine, Adrenergic alpha-agonists, Antidepressant, Depression, Major depressive disorder, Treatment resistant-depression

## Abstract

Depressive disorders (DD) are common, and their prevalence is expected to rise over the next decade. Depressive disorders are linked to significant morbidity and mortality. The clinical conundrum of depressive disorders lies in the heterogeneity of their phenomenology and etiology. Further, the currently available antidepressants have several limitations, including a delayed onset of action, limited efficacy, and an unfavorable side effect profile. In this review, Dexmedetomidine (DEX), a highly selective and potent α2-adrenergic receptor (α2-AR) agonist, is proposed as a potentially novel antidepressant with multiple mechanisms of action targeting various depression pathophysiological processes. These mechanisms include modulation of the noradrenergic system, regulation of neuroinflammation and oxidative stress, influence on the Brain-Derived Neurotrophic Factor (BDNF) levels, and modulation of neurotransmitter systems, such as glutamate. The review begins with an introduction before moving on to a discussion of DEX's pharmacological features. The pathophysiological and phenomenological targets of DD are also explored, along with the review of the existing preclinical and clinical evidence for DEX's putative anti-depressant effects. Finally, the review ends by presenting the pertinent conclusions and future directions.

## 1 INTRODUCTION

Depressive disorders (DD) are a class of pleomorphic mood disorders that arise from genetic, neurobiological, and psychosocial factors interacting to produce a variety of symptomatologic expressions. DD include major depressive disorder (MDD), persistent depressive disorder (PDD), and other depressive disorders, which are regarded to be among the most common mental health conditions, with an estimate of over 300 million people and approximately 4.4% of the world's population [1]. DD are debilitating mental conditions that diminish the quality of life and severely affect individuals’ social and occupational function. They have been identified as the major cause of global disability by the World Health Organization (WHO) [56]. In addition, DD are associated with suicidal ideation, self-harm, and attempted and completed suicide [2]. Despite the severity of these disorders, there are till many challenges and unmet needs related to their treatment.

One of the most challenging clinical presentations of DD is treatment-resistant depression (TRD). Despite the lack of consensus on the definition, TRD can be defined as a condition in which a patient's depression treatment fails to achieve remission after two or more treatment attempts with a sufficient dose and duration [3]. The prevalence of TRD was estimated to be 30.9% of all patients with medication-treated MDD in the United States, with a $43.8 billion annual national economic burden [4]. Patients with TRD generally experience severe morbidity, suicidal thoughts and behavior, and medical complications secondary to depression-related exacerbation of physical health conditions that are linked with higher rates of hospitalization along with reduced productivity in working individuals and higher unemployment rates [4]. Therefore, effective treatment of TRD has the potential to yield significant societal and economic benefits. Currently, there are only three treatment modalities approved by the U.S Food and Drug Administration (FDA) for the treatment of TRD: electroconvulsive therapy (ECT) [5]; ketamine intravenous infusion; intra-nasal esketamine in combination with an oral anti-depressant [6]; and olanzapine-fluoxetine combination [7]. Besides limited response to treatment, available antidepressants have other shortcomings, including disease-modifying effects and not targeting certain symptom dimensions, such as sleep disturbance [8]. Sleep disturbance, which includes trouble initiating and maintaining sleep as well as a subjective experience of poor sleep quality, is a clinical sign of DD, and current antidepressants have limited effectiveness in treating sleep problems. In fact, several antidepressants worsen sleep by causing or worsening insomnia, daytime sleepiness, and/or sedation [9]. Many patients are non-compliant with antidepressant therapy due to its unwanted adverse effects on sleep [10]. Therefore, developing an anti-depressant that improves symptoms of depression while also alleviating sleep problems would increase patients’ compliance with therapy and ultimately increase the remission rates.

Subtypes of DD may be associated with poor response to treatment. One of these subtypes is MDD with anxious distress (*i.e.*, MDD episode accompanied by at least two or more anxiety symptoms not meeting the criteria of generalized anxiety disorder). Patients with this depression subtype have an overall poor treatment outcome to a wide range of antidepressant agents with a small reduction of symptoms, a large burden of a side-effect, and a long time to recovery [11]. Another subtype is MDD with mixed episodes (*i.e.*, MDD episode accompanied by at least 3 manic or hypomanic symptoms not meeting the criteria of bipolar disorder). Only a few studies have investigated pharmacological therapies for this subtype, with the majority of them focusing on antipsychotics, despite scant evidence of their efficacy. As a result, exploring new pharmacological treatment modalities is of vital importance [12].

Besides, most pharmacological treatments for MDD do not produce a rapid onset of antidepressant effects. Selective serotonin reuptake inhibitors (SSRI) and serotonin and norepinephrine reuptake inhibitors (SNRI) are usually the first-line and the most commonly prescribed pharmacological therapy. However, initial treatment with SSRI and/or SNRI takes at least 4 weeks to attain response and 6 weeks to attain remission, and occasionally remission can take up to 12 weeks or longer. Therefore, there is an urgent need for developing an antidepressant with a rapid onset occurring within hours to days instead of weeks to months to prevent the major depressive episode’s deleterious psychosocial effects and the long-term neurobiological effects on the patient’s brain function [13]. Currently, the U.S FDA has approved only three modalities with rapid anti-depressant efficacy, the ECT [5], the intra-nasal esketamine spray [6], and the brexanolone injection/infusion for post-partum depression [14].

Overall, MDD is a prevalent and detrimental clinical entity with heterogeneity in etiological and phenomenological aspects. The available treatments have several caveats which call for exploring new treatment modalities. In the current review, the authors present dexmedetomidine as a potential antidepressant agent. We will provide a brief review of the pharmacological properties of this medication and review the recent evidence in the literature. We will also discuss what could be the advantages of dexmedetomidine compared to traditional antidepressants, including rapid onset of antidepressant effect, improvement of sleep-related symptoms, and overall effectiveness. This paper will review the preclinical and clinical research supporting dexmedetomidine's therapeutic effects in MDD.

This review included clinical and preclinical publications investigating and/or indicating DEX antidepressant potentials between January 1999 and May 2022. The team searched major databases; PubMed, SCOPUS, Cochrane Database; MEDLINE^TM^, and EMBASE^TM^. The Search terms were: 1) “Dexmedetomidine” or “Dexdor” or “Precedex” or “Dexdomitor”; 2) “depressive symptoms”, “depressive illness”, “depressive behaviors”, “major depression”, “MDD”, “unipolar depression”, “postpartum depression”, “postpartum depressive symptoms”. There were no initial restrictions imposed with regards to language or study design. The studies were included in the review based on the following criteria: fully peer-reviewed, focused on DEX antidepressant potentials from clinical, preclinical, and mechanistic perspectives. We included studies that were reported in English or translated into English. At first, given the limited references on the subject under investigation, the authors performed a scoping review instead of a formal systematic review. Then, for final inclusion in the review, we screened the abstracts of potentially eligible publications, removing duplicates and unrelated articles. The included research articles provided the pertinent data and components for this review. In the current synthesis, the authors presented the evidence deductively and inductively.

### 1.1 Dexmedetomidine a Selective Alpha 2-Adrenoceptor Agonist

Dexmedetomidine is a selective and potent α_2_-adrenoceptor agonist. It is the active dextro-isomer of medetomidine. It was approved for intravenous (IV) sedation of mechanically ventilated adult patients in the intensive care unit (ICU) for up to 24 hours by the U.S FDA in 1999. An additional indication was approved in the US in 2008 [15], enabling DEX use in sedating non-intubated patients prior to and/or during surgical and other procedures. DEX was registered in the European Union in 2011 for the sedation of adult ICU patients who required a level of sedation that allowed them to respond to verbal stimulation [16].

### 1.2 Pharmacokinetics of Dexmedetomidine

The intravenous route (IV) is the standard method to administer DEX. Intramuscular [17], spinal [18], epidural [19], buccal [20], peripheral nerve block [21] and intra-nasal [20] delivery are among the various methods of administration being investigated by researchers. DEX has an onset of action of approximately 15 minutes after being administered intravenously, and with continuous IV perfusion, peak concentrations are frequently reached within 1 hour. DEX has a rapid distribution phase, a steady-state volume of distribution of 118 L, a distribution half-life of 6 minutes, and a clearance of 39 L.h^-1^ in adults, and overdose ranges of 0.2-0.7 μg.kg^-1^.h^-1^. It also has an elimination half-life of 2.0 to 2.5 hours[22]. DEX binds to albumin and can pass the Blood-Brain Barrier (BBB) and placenta, but its teratogenic effects have not yet been fully explored. DEX is metabolized in the liver through glucuronide conjugation uridine 50 diphospho-glucuronosyltransferase (UGT2B10, UGT1A4) and biotransformation mediated by the cytochrome P450 (CYP2A6). About 95% of the metabolites are excreted renally, whereas just 4% are excreted fecally [23].

### 1.3 Pharmacodynamics of Dexmedetomidine

When administered *via* IV bolus, DEX leads to an increase in blood pressure and a significant drop in heart rate due to a high peak in plasma concentration. This is assumed to be caused by the activation of α_2_-receptors in the vascular smooth muscles, which causes peripheral vasoconstriction and hypertension. This is accompanied by a quick reduction in heart rate, which is thought to be caused by the baroceptor reflex . The vasoconstriction subsides within a few minutes as DEX plasma concentrations fall. Moreover, DEX activates α_2_-receptors in vascular endothelial cells, resulting in vasodilation. This results in a hypotensive phase due to presynaptic α_2_-adrenoreceptors limiting sympathetic catecholamine release and enhanced vagal activity [24,25]. DEX's anxiolytic and analgesic effects are assumed to be mediated mainly through its ability to bind to α_2_-receptors in the central nervous system and spinal cord [26,27]. DEX's sedative and hypnotic effects are assumed to be mediated through central pre-synaptic and postsynaptic α_2_-receptor activation in the locus coeruleus [27].

### 1.4 Adverse Effects of Dexmedetomidine

The majority of dexmedetomidine-related side effects occur during or shortly after a loading dose. Nausea, hypoxia, hypertension, hypotension, atrial fibrillation, and bradycardia are common side effects of a loading infusion, and they are all linked to the loading dose and infusion rate [28]. The incidence of these hemodynamic effects can be prevented by omitting bolus loading or slow bolus loading [29]. In addition, atrioventricular block of the first or second degree can result from an overdose [30]. Meanwhile, Selective serotonin reuptake inhibitors (SSRIs) have several side effects. These include gastrointestinal disturbances like nausea and diarrhea, sexual dysfunction, such as decreased libido and difficulty achieving orgasm, sleep disturbances like insomnia or drowsiness, and weight changes (either gain or loss). Other potential side effects include restlessness, headaches, sweating, dry mouth, dizziness, and lightheadedness [31]. Therefore, DEX shows possibly a more favorable side effect profile.

### 1.5 Depressive Disorders’ Pathophysiological Targets for Dexmedetomidine

In this section, the authors highlight the pathophysiological processes of depressive disorders which can be targeted by dexmedetomidine, including the immuno-inflammatory mechanisms, the neuro-progression hypothesis, and the role of the Brain-Derived Neurotrophic Factor (BDNF), and the involvement of the adrenergic system in depressive disorder’s pathogenesis.

**Fig. (1):**
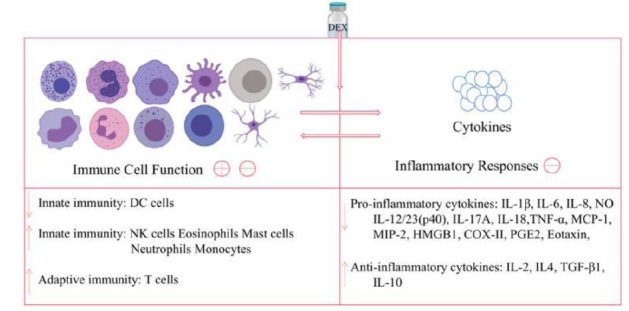
Effects of dexmedetomidine on immune cells and inflammatory cytokines. Available online under a CC BY 4.0 license. [38].

Firstly, the immune system and the inflammatory process have been implicated in the pathogenesis of depressive disorders, and various studies have found that pro-inflammatory cytokines play a key role in the development of depression. Several reports indicated that patients with depressive disorders have higher levels of pro-inflammatory cytokines, such as C-reactive protein (CRP), interleukin (IL)-1, (IL)-6, and tumor necrosis factor-alpha (TNF-alpha) in their plasma than healthy individuals [32]. Moreover, individuals with inflammatory-related diseases, such as asthma, arthritis, obesity, diabetes, and coronary artery disease, are more likely to have co-morbid MDD supporting a relationship between inflammation-associated depression etiopathogenesis [33]. With the above being highlighted, DEX has been found to have anti-inflammatory properties and can greatly reduce the production of cytokines, such as IL-1, IL-6, TNF-alpha, and Nuclear Factor Kappa B (NF-kappa-B) across various studies [34,35]. A meta-analysis by Bo *et al.* found that when DEX was given as an addition to general anesthesia, it significantly reduced postoperative serum IL-6, IL-8, and TNF-alpha levels both immediately after surgery and on the first postoperative day [36]. Another study also found that adjuvant administration of DEX during surgery decreased CRP and leukocyte count on the first postoperative day (Fig. **1**) [37].

Secondly, the neuroprotective and neurotrophic signaling mechanisms are required for the growth, maturation, and survival of neurons and glia in the adult brain in general. Concerning depressive disorders, findings from preclinical and translational studies provide strong evidence that neurotrophic factors and neuroprotective pathways are disrupted [39]. Nonetheless, data have shown that the administration of dexmedetomidine can stimulate neurotrophic and neuroprotective pathways. DEX exerts a neuroprotective effect and helps to prevent neuronal damage. First off, it has the potential to block the release of excitatory neurotransmitters, such as glutamate, a known potential target for ketamine. DEX decreases glutamate’s release *via* several proposed mechanisms, including reducing the release of catecholamine and blocking the mitogen-activated/extracellular signal-regulated kinase pathway and voltage-dependent calcium channels.

Second, by its anti-apoptotic activity, which protects against oxygen-glucose deprivation-induced injury, and finally, through its ability to regulate inflammatory mediators (IL-1, IL-6, TNF-alpha, and NF-kappa-B) and reduce oxygen free-radical generation [35]. There are several randomized clinical trials suggesting DEX neuroprotective properties. Xiahong Luo *et al.* concluded that intra-operative DEX administration could reduce brain damage caused by craniotomy in glioma patients; they experienced brain protection by achieving hemodynamic stability of heart rate and mean arterial pressure (MAP), attenuated inflammation, and inhibited free radicals generation [40]. Ashish Bindra et al. [41] concluded that intra-operative DEX administration might play a role in cerebroprotection by attenuating the release of biomarkers that cause cerebral insults, such as neuron-specific enolase (NSE) and S100b in chronic temporal lobe epilepsy patients during surgery for this disease.

Thirdly, Brain-Derived Neurotrophic Factor (BDNF) is a neurotrophic factor that promotes neuronal plasticity, maintenance, and survival, along with neurotransmitter regulation. A meta-analysis study revealed lower serum BDNF levels in depressed subjects compared to healthy subjects and found higher BDNF levels post anti-depressant treatment [42]. Moreover, post-partum studies have demonstrated decreased levels of BDNF and BDNF receptor levels in the hippocampus of patients with depression and suicide victims [43,44]. A systemic review and meta-analysis by Andre *et al.* suggested a correlation between anti-depressant treatment indexed to blood BDNF levels and improvement of depressive symptoms through an increase in neuronal neuroplasticity [45]. DEX exhibits a neurotrophic ability by enhancing the BDNF4 and BDNF5 transcription and BDNF levels in astrocytes through the extra-cellular signal-regulated kinase-dependent pathway [35]. A double-blind, randomized, and placebo-controlled trial suggested that intravenous DEX infusion during carotid endarterectomy improves cognition by increasing BDNF concentration [46].

Lastly, the adrenergic system is also implicated in the pathogenesis of depressive disorders because it was shown that the sympathetic adrenergic system is overactive or hyperresponsive in depression [47]. Numerous studies in humans [48] and in animal models [49] indicate that the hyperactive adrenergic system can be involved in the pathophysiology of depression. Stress increases sympathetic nervous system activity while decreasing parasympathetic nervous system activity. This results in high levels of epinephrine and norepinephrine and low levels of acetylcholine. In turn, this can lead to an increase in pro-inflammatory cytokines like TNF-alpha, IL-1, and IL-6, as well as a reduction in anti-inflammatory cytokines like IL-10, resulting in a state of inflammation. An imbalance between neuroprotective and neurotoxic kynurenine metabolites occurs as a result of the inflammatory state. This can lead to neurodegenerative alterations in the brain, making the brain more susceptible to depression [50]. On the other hand, dexmedetomidine treatment was found to be associated with a low level of kynurenine metabolism in patients with acute brain dysfunction. Nevertheless, further studies are needed to examine the mechanisms by which dexmedetomidine interferes with the kynurenine pathway.

**Fig. (2):**
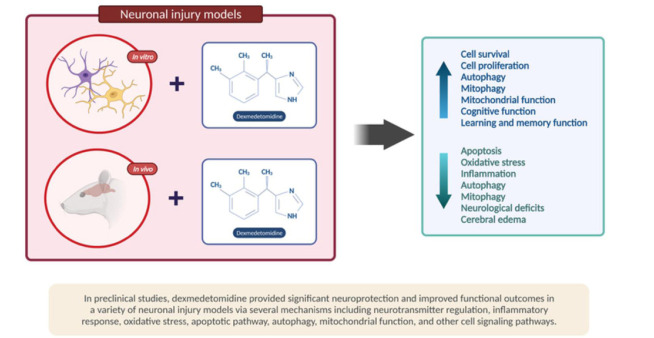
Summary of the potential neuroprotective mechanisms of dexmedetomidine in brain injury. Available online ubder a John Wiley and Sons and Copyright Clearance Center (CCC) [52].

Besides, studies in animal models show that dexmedetomidine decreases the release of catecholamines in nerve endings, lowering sympathetic nervous system activity and reducing neuronal damage (Fig. **2**) [51].

### 1.6 Preclinical Data on Dexmedetomidine Antidepressant Potential

This section explores DEX's antidepressant effect in animal models of depression. It highlights the specific neurotransmitters' effects, as well as neuro-regenerative and cellular processes involved in promoting recovery from depression.

The first animal research demonstrating DEX's antidepressant effects was performed by Moon *et al.* [53]. It was conducted on mice with sleep-deprived induced depressive behaviors. The study revealed that DEX alleviated sleep deprivation-induced depressed behaviors within a short time period of about six days by enhancing 5-HT synthesis and lowering dopamine production while up-regulating the D1 dopamine receptor. Moreover, DEX was found to activate dopaminergic neurons in the ventral tegmental area of adult mice and increase dopamine concentrations in the related forebrain projection areas *via* α_2_-receptor-dependent mechanisms, which may contribute to rapid arousability during DEX sedation. Based on these findings, DEX may have a potential application in the treatment of depression *via* dopaminergic modulation [54].

Additionally, another animal study done by Xin Gao *et al.* [55] revealed that DEX achieved cognitive neuroprotection by enhancing memory and decreasing learning deficits brought about by electroconvulsive therapy in depressed mice. In particular, DEX cognitive neuroprotection was achieved by suppressing excessive NMDA Receptor Subunit 2B (NR2B) up-regulation.

Besides, Ji *et al.* [56] suggested that DEX protects rats with post-traumatic stress disorder against anxiety-like behaviors and spatial cognitive dysfunctions. In addition, it reported that the fear-conditioned memory was weakened. Moreover, in a postnatal day-7 rat experimental model conducted by Pancaro *et al.* [57] aimed at evaluating and comparing the effects of ketamine and DEX on brain cell degeneration and apoptosis, DEX caused considerable cellular degradation in primary sensory brain regions, but non-significant changes in limbic regions. In contrast, ketamine caused a significant limbic injury. Given DEX's limbic preservation function, this study could suggest DEX’s efficacy in emotional, learning, and memory modulation.

In regards to DEX’s neuro-regenerative effect, a recent study by Xu *et al.* [58] conducted among mice found that DEX reduced depression induced by chronic neuropathic pain by promoting cellular proliferation and neuronal differentiation in the dentate gyrus region of the hippocampus. Jimenez-Tellez *et al.* [59] studied the effects of DEX on cellular homeostasis, viability, growth, and synaptic assembly in rat cortical neurons *in vitro*, as well as in postnatal day 7 pups to examine whether DEX exposure *in vivo* could impair learning and memory. The researchers found that DEX exposure did not affect cell viability or neuronal ability to initiate growth; rather, DEX-exposed cells had more branching per neural process than their control counterparts, with no significant difference in the overall number of synaptic puncta per cell. Furthermore, DEX-treated cells maintained a healthy ROS production pattern. Lastly, when DEX was given to young pups, it did not impair learning or memory, indicating that it is safe for clinical use.

Moreover, these findings highlight DEX's capacity to address DD's cognitive dimensionality.

### 1.7 Clinical Data on Dexmedetomidine Antidepressant Potential

This section focuses on DEX clinical trials in people who have depressive symptoms. Antidepressant evidence from research on patients with different psychiatric diseases is also included.

First, a randomized, double-blind controlled study by Shams *et al.* [60] compared induction with ketamine-propofol, along with DEX (ketofol-dex group) to ketamine-propofol only (ketofol group) on patients with depression undergoing ECT. In this trial, the researchers found that the ketofol-dex group experienced a more acceptable decrease in heart rate and blood pressure, less agitation, longer mean seizure duration, more effective anti-depression, good analgesia, sedation, and higher patient satisfaction than the ketofol group. This RCT backs up DEX's potential rapid antidepressant properties. To begin, the ketofol-DEX group reported reduced depressive scores on the Hamilton depression rating scale on day 1 after ECT. Second, the ketofol-DEX group had a longer mean seizure duration, indicating a greater antidepressant potential. While the authors of the study indicated that the ketofol-dex combination had an antidepressant effect, the trial only lasted for five days. This made determining the antidepressant efficacy over a longer period difficult. Furthermore, because of ECT's well-known and substantial efficacy, attempting to tease out the biological efficacy of DEX on depressive symptoms in patients undergoing ECT is difficult.

Moreover, Yu *et al.* [61] investigated the efficacy of DEX in the prevention and treatment of postpartum depression symptoms following cesarean section in a randomized, double-blind, placebo-controlled trial. The findings of the study concluded that DEX administration in the early postpartum period could significantly reduce the incidence of postpartum blues, postpartum depressive disorders, and postpartum self-harm ideation, improve postpartum sleep quality, and improve maternal postoperative analgesia. It has been noted that this study excluded patients having a history of psychosis and significant complications linked with chronic conditions. A related study aimed to investigate the association between α2A adrenergic receptor (α2AAR) gene polymorphisms and postpartum depressive symptoms (PDS). The researchers focused on the application of dexmedetomidine in improving maternal sleep and reducing the incidence of PDS. A total of 568 cesarean-section patients were enrolled, with 18.13% experiencing PDS (103 with PDS, 465 without PDS). PDS was diagnosed using the Edinburgh Postpartum Depression Scale score at 42 days after delivery, and the researchers stated that DEX's α2-receptor activation is responsible for the considerable reduction in postpartum depressive symptoms incidence. Because they found that women with the *α_2A_AR* rs13306146 AA polymorphism had a significantly higher risk of postpartum depressive symptoms, this genotype results from transcriptional *α_2A_AR* inhibition, and it increases neuronal responses to excitatory stimuli, consequently increasing depression risk and vulnerability [62].

Furthermore, Dong *et al.* [63] performed a prospective randomized controlled clinical trial to investigate whether prophylactic nocturnal DEX reduces the risk of post-intensive care syndrome (PICS) post-cardiac surgery. Following a severe illness, PICS refers to new or worsening physical, mental, and cognitive disability. According to the study, PICS incidence in the DEX group was significantly lower at the 6-month follow-up than in the placebo group (21.5% *vs*. 31.1%, OR 0.793, 95% CI 0.665–0.945; p = 0.014) and it attributed to the significant reduction in psychological impairment. In this RCT, depression and anxiety were chosen as representative features for psychological impairment, and they were evaluated by the Zung Self-Rating Depression Scale (SDS) and Zung Self-Rating Anxiety Scale (SAS). Psychological impairment by SDS and SAS in the DEX group was (18.7%) and in the placebo group was (26.8%), P value of 0.029, signifying the sustained antidepressant effects of DEX in the longer term. However, because this study only included patients undergoing cardiac surgery, it is less acceptable for clinical practice when managing patients with different conditions, limiting the generalizability of the findings. Furthermore, the patients were allowed to receive extra sedatives if necessary. As a result, some of the relief with PICS could be attributed to these additional sedatives.

In addition to that, a study where patients with chronic intractable insomnia were treated with Patient-Controlled Sleep (PCSL), and DEX was used instead of traditional analgesics in Patient-Controlled Analgesia (PCA), it was found that PCSL could improve the subjective assessment of sleep in 12 out of 15 patients with chronic intractable insomnia immediately after therapy, and 7 out of 12 patients had consistently improved sleep quality over a 6-month period. The Pittsburgh Sleep Quality Index was used to assess subjective sleep quality. Moreover, the Hamilton Anxiety Scale (HAMA), Hamilton Depression Scale (HAMD), and Chinese versions of the Symptom Checklist (SCL-90) were used to assess the patients for anxiety, depression, and psychological problems. Prior to PCSL treatment, the study found that all of the patients experienced anxiety and depression, and six of them had mild psychological problems. The HAMA and HAMD scores were significantly reduced immediately post-treatment and at follow-up periods (p<0.05) [64]. However, because of the small sample size, the study's generalizability is limited (Table **1**).

Due to its safety and neuro-modulatory effects, Goswami *et al.* [65] recently utilized DEX while conducting drug-assisted psychiatric interviews. In this case series, DEX outperformed thiopentone in terms of achieving a level of conscious sedation that allowed the subjects to be interviewed. Moreover, the recovery went more smoothly with DEX, and the interviewer’s satisfaction was better. Although only one of the two cases, who suffered from dissociative symptoms, showed clinical improvement. This study indicates that DEX infusion in psychiatric settings with anesthesiologist supervision is feasible.

Quite recently, the U.S. FDA approved an orally absorbed, self-administered, sublingual film DEX known as (Igalmi) by BioXcel Therapeutics for the acute treatment of agitation associated with bipolar I or II disorder or schizophrenia in adults. The approval is based on results from two phase 3 randomized, double-blinded, placebo-controlled trials that tested DEX for the acute treatment of agitation associated with schizophrenia (SERENITY I) or bipolar type 1 or type 2 disorder (SERENITY II). Treatment with a sublingual film formulation of 120 μg or 180 μg, compared to placebo, resulted in a significantly greater reduction in the agitation score measured by the Positive and Negative Syndrome Scale-Excited Component (PEC) at 2 hours in patients with bipolar disorder type 1 or 2 who were experiencing mild to moderate agitation based on baseline PEC score (both doses P < .001 *vs* placebo). Treatment effects began as early as 20 minutes for both doses [66]. Nevertheless, based on their baseline PEC score, the patients in this study had mild to moderate agitation. Moreover, the level of cooperation required to administer a medication sublingually limits the generalizability of these findings to patients who are able or willing to self-administer this treatment.

Finally, in a study aimed at evaluating the safety and efficacy of two small-dose infusions of DEX, the researchers assessed sedation, analgesia, cognition, and cardiorespiratory function, and administration of DEX at a dose of 0.1-0.2 mg/kg/hr over a 40-minute period was found to be safe and well-tolerated, with no notable side effects observed [67]. Notably, a positive biological response was observed within the first week of treatment, as assessed by the Montgomery–Åsberg Depression Rating Scale (MADRS).

## CONCLUSION

To summarize, we suggest the antidepressant effect and safety of DEX deserve further study. This medication could be potentially useful in the treatment of depressive disorders based on DEX's pharmacodynamics, the available preclinical evidence, and the promising clinical data, such as its antidepressive effect [60, 64], tolerability [60], and rapid onset of action [66]. This proposition could be explored in a variety of clinical settings. For instance, DEX’s putative antidepressant effect and established anxiolytic action make it a potential option for patients with DD who are experiencing anxious distress. Also, due to its efficacy on insomnia, DEX could be used to treat individuals diagnosed with depressive disorders who experience prominent sleep difficulties. However, several pertinent questions must be addressed, including which subpopulation of patients with DD would benefit the most? What is the ideal setting? What is the recommended dosage? What should the duration of the infusion be? How often should it be administered? Patients with treatment-resistant DD without contraindications to DEX should be the first subgroup to be included in clinical trials with this medication. The protocol used by Hall *et al.* [67] could serve as a starting point for designing pilot studies, open-label studies or randomized controlled trials, using depressive symptom severity as the primary outcome measure, while monitoring the tolerability and safety of short-term and long-term use of DEX.

## AUTHORS’ CONTRIBUTION

TA, MA, and RF contributed to the conceptualization. TA, MA, RF also contributed to the literature review. TA and MA contributed to the writing. RF contributed to review and Editing.

## Figures and Tables

**Table 1:** Summary of clinical studies of antidepressant activity of dexmedetomidine.

**Study Authors and Publication Year/Refs.**	**Study Design**	**Results**
Yu *et al.* 2019 [61]	This study followed a randomized, double-blind, placebo-controlled design. A total of 600 women who were scheduled to have elective cesarean delivery with spinal anesthesia were divided randomly into two groups: the control group, which received a normal saline infusion after delivery, and patient-controlled intravenous analgesia (PCIA) with sufentanil, and the DEX group, which received a dexmedetomidine (DEX) infusion of 0.5 μg/kg after delivery and PCIA with a combination of DEX and sufentanil.	The prevalence of postpartum blues and postpartum depressive symptoms (PDS) was significantly lower in the DEX group compared to the control group (5.0% *vs*. 14.1%, p<0.001; 5.7% *vs*. 16.3%, p<0.001, respectively), particularly among women who had antenatal depression or moderate stress during pregnancy. Furthermore, the Edinburgh Postnatal Depression Scale (EPDS) score at postpartum days 7 and 42 was significantly lower in the DEX group compared to the control group (4.23 ± 4.37 *vs* 1.93 ± 3.36, p<0.001; 4.68 ± 4.78 *vs* 1.99 ± 3.18, p<0.001, respectively). Additionally, the incidence of postpartum self-harm ideation at postpartum days 7 and 42 was lower in the DEX group compared to the control group (1.1% *vs* 4.0%, p=0.03; 0.4% *vs* 2.9%, p=0.04, respectively). The DEX group also reported better pain scores and sleep quality compared to the control group (p<0.001).
Duan *et al* 2021 [62]	This study examined the association between α2A adrenergic receptor (α2AAR) gene polymorphisms and postpartum depressive symptoms (PDS). The researchers investigated the use of DEX to enhance maternal sleep quality and decrease the occurrence of PDS. A total of 568 patients who had undergone cesarean-section delivery were included in the study.	18.13% experienced PDS (103 with PDS, 465 without PDS). PDS was diagnosed using the Edinburgh Postpartum Depression Scale score 42 days after delivery. The substantial decrease in the occurrence of postpartum depressive symptoms can be attributed to the activation of α2-receptors by DEX. The study revealed that women with the α2A adrenergic receptor (α2AAR) rs13306146 AA polymorphism had a significantly elevated risk of experiencing postpartum depressive symptoms. This specific genotype is associated with transcriptional inhibition of α2AAR, leading to heightened neuronal responses to excitatory stimuli, which ultimately increases the risk and vulnerability to depression.
Dong *et al* 2020 [63]	This study followed a double-blind, randomized, prospective, and placebo-controlled design. A total of 508 patients who were undergoing cardiac surgery were randomly assigned to either the dexmedetomidine group or the placebo group (which received normal saline). Dexmedetomidine or a similar amount of saline was administered through infusion at a rate of up to 1.2 μg/kg/h until the Richmond Agitation-Sedation Scale (RASS) remained between -1 and 0.	The incidence of post-intensive care syndrome (PICS) at the 6-month follow-up was significantly lower in the dexmedetomidine group (54 out of 251, 21.5%) compared to the placebo group (80 out of 257, 31.1%) (odds ratio [OR] 0.793, 95% confidence interval [CI] 0.665-0.945; p = 0.014). Furthermore, the dexmedetomidine group showed a significant reduction in psychological impairment compared to the placebo group (18.7% *vs*. 26.8%, OR 0.806, CI 0.672-0.967, p = 0.029).
Shams *et al.* 2014 [60]	This study followed a randomized, double-blind design. A total of 40 patients were slated to receive electroconvulsive therapy (ECT) as a treatment for depression. Before administering anesthesia with a combination of ketamine and propofol (ketofol), patients received either dexmedetomidine diluted with 10 ml of 0.9% saline at a concentration of 0.5 μg/kg (ketofol-DEX group) or a 10 ml infusion of 0.9% saline alone was given intravenously over a duration of 10 minutes (ketofol group).	The duration of motor seizures was significantly shorter in the ketofol group compared to the ketofol-dex group (35.8 ± 6.6 seconds *versus* 38.9 ± 4.9 seconds). Additionally, the total amount of ketofol used was significantly lower in the ketofol-dex group compared to the ketofol group (78.5 ± 10.8 mg *versus* 90 ± 13.2 mg). The number of patients experiencing agitation with a score greater than 2 was significantly lower in the ketofol-DEX group (1.4%) compared to the ketofol group (8.6%). Furthermore, there was a significant decrease (P = 0.000) in mean arterial pressure (MAP) and heart rate (HR) in the ketofol-DEX group compared to the ketofol group at 20, 30, and 40 minutes for MAP, and at 10, 20, 30, and 40 minutes for HR. In terms of the scores obtained from the Hamilton Depression Rating Scale (HDRS), both groups showed a significant decrease (P ≤ 0.01) from 1 to 5 days following ECT treatment compared to the scores before the ECT. Furthermore, a significant difference between the two groups was observed only after the first day of the initial ECT session, day 1 post ECT the ketofol-DEX group had lower depressive scores compared to the ketofol group.
An *et al.* 2020 [64]	This study followed a single-center, single-arm pilot design. A total of 20 patients with chronic intractable insomnia underwent a treatment known as Patient-Controlled Sleep (PCSL), in which traditional analgesics in Patient-Controlled Analgesia (PCA) were replaced with dexmedetomidine (DEX). The patients were assessed using the Pittsburgh Sleep Quality Index (PSQI), Symptom Checklist 90 (SCL-90), Hamilton Anxiety Scale (HAMA), and Hamilton Depression Scale (HAMD) both before and after the treatment.	15 patients successfully completed the treatment protocol. Following the therapy, an immediate improvement in sleep quality was observed in 12 out of the 15 patients. Among these 12 patients, 7 continued to experience sustained improvements in sleep quality during the 6-month follow-up period. Additionally, both the Hamilton Anxiety Scale (HAMA) and Hamilton Depression Scale (HAMD) scores showed significant reductions immediately after the treatment and at subsequent follow-up assessments (p<0.05).

## LIST OF ABBREVIATIONS

BBB: = Blood-Brain Barrier
BDNF: = Brain-Derived Neurotrophic Factor
DD: = Depressive Disorders
DEX: = Dexmedetomidine
ECT: = Electroconvulsive Therapy (ECT)
ICU: = Intensive Care Unit
IV: = Intravenous
HAMA: = Hamilton Anxiety Scale
HAMD: = Hamilton Depression Scale
MADRS: = Montgomery–Åsberg Depression Rating Scale
MDD: = Major Depressive Disorder
PCA: = Patient-controlled Analgesia
PICS: = Post-intensive Care Syndrome
PCSL: = Patient-controlled Sleep
SCL-90: = Chinese Versions of the Symptom Checklist
SSRI: = Selective Serotonin Reuptake Inhibitor
SNRI: = Serotonin and Norepinephrine Reuptake Inhibitor
TRD: = Treatment-resistant Depression
WHO: = World Health Organization
